# Copper-Dependent Trafficking of the Ctr4-Ctr5 Copper Transporting Complex

**DOI:** 10.1371/journal.pone.0011964

**Published:** 2010-08-04

**Authors:** Raphaël Ioannoni, Jude Beaudoin, Alexandre Mercier, Simon Labbé

**Affiliations:** Département de Biochimie, Faculté de médecine et des sciences de la santé, Université de Sherbrooke, Sherbrooke, Canada; University College Dublin, Ireland

## Abstract

**Background:**

In *Schizosaccharomyces pombe*, copper uptake is carried out by a heteromeric complex formed by the Ctr4 and Ctr5 proteins. Copper-induced differential subcellular localization may play a critical role with respect to fine tuning the number of Ctr4 and Ctr5 molecules at the cell surface.

**Methodology/Principal Findings:**

We have developed a bimolecular fluorescence complementation (BiFC) assay to analyze protein-protein interactions *in vivo* in *S. pombe*. The assay is based on the observation that N- and C-terminal subfragments of the Venus fluorescent protein can reconstitute a functional fluorophore only when they are brought into tight contact. Wild-type copies of the *ctr4^+^* and *ctr5^+^* genes were inserted downstream of and in-frame with the nonfluorescent C-terminal (VC) and N-terminal (VN) coding fragments of Venus, respectively. Co-expression of Ctr4-VC and Ctr5-VN fusion proteins allowed their detection at the plasma membrane of copper-limited cells. Similarly, cells co-expressing Ctr4-VN and Ctr4-VC in the presence of Ctr5-Myc_12_ displayed a fluorescence signal at the plasma membrane. In contrast, Ctr5-VN and Ctr5-VC co-expressed in the presence of Ctr4-Flag_2_ failed to be visualized at the plasma membrane, suggesting a requirement for a combination of two Ctr4 molecules with one Ctr5 molecule. We found that plasma membrane-located Ctr4-VC-Ctr5-VN fluorescent complexes were internalized when the cells were exposed to high levels of copper. The copper-induced internalization of Ctr4-VC-Ctr5-VN complexes was not dependent on *de novo* protein synthesis. When cells were transferred back from high to low copper levels, there was reappearance of the BiFC fluorescent signal at the plasma membrane.

**Significance:**

These findings reveal a copper-dependent internalization and recycling of the heteromeric Ctr4-Ctr5 complex as a function of copper availability.

## Introduction

Given the physiological demands for copper across the biome, both unicellular and multicellular organisms share the requirement for acquiring sufficient levels of copper for cell development and proliferation [1]. Copper serves as a catalytic or a structural cofactor for many of the enzymes that are intimately linked to essential cellular functions, including the ones involved in respiration, antioxidant defense, iron transport and the bioactivation of enzymes and hormones [2], [3]. Paradoxically, when present in excess, copper becomes a potent cytotoxin due to its ability to react with hydrogen peroxide in a reaction that produces detrimental hydroxyl radical [4]. Therefore, it is critical that organisms maintain homeostatic mechanisms to acquire sufficient amounts of copper, yet prevent its accumulation to toxic levels.

Eukaryotes from yeast to humans use the copper transporter (Ctr) family of transporters for uptake of copper across the plasma membrane [5]. Although Ctr amino acid sequences exhibit a limited overall sequence homology between family members, most of the Ctr transporters share the following overall features. An extracellular N-terminal region of variable length contains methionine residues arranged as MX_2_M and/or MXM motifs (denoted Mets motifs) [5]. A first transmembrane span is connected to a second transmembrane span by an intracellular loop of variable length. Transmembrane spans 2 and 3 are joined by a short linker region. Transmembrane span 2 contains a highly conserved MX_3_M motif that is essential for function in copper transport, whereas transmembrane span 3 possesses a conserved GX_3_G motif that is required for the trimeric assembly of Ctr molecules [6], [7]. An intracellular C-terminal tail of variable length possesses, in general, some cysteine and histidine residues that may be involved in copper binding [1].

The fact that sequences of Ctr proteins vary considerably in both length and composition may explain the reason why a number of studies have reported various mechanisms for post-transcriptional regulation of these proteins [8]–[11]. Studies in the baker's yeast *Saccharomyces cerevisiae* have shown that copper is taken up through two high affinity copper transporters, Ctr1 and Ctr3 [12]–[14]. Although Ctr1 and Ctr3 are functionally redundant, these two plasma-membrane proteins mediate copper uptake independently of each other [13]. At the post-transcriptional level, Ctr3 is differently regulated, as compared to Ctr1, in terms of copper levels [8], [14]. Ctr3 steady-state levels at the cell surface are stable under both low and high copper concentrations, whereas Ctr1 has been reported to undergo different modes of regulation in response to exogenous copper [8], [14]–[16]. One study indicated that Ctr1 is subjected to two forms of post-translational regulation: endocytosis and proteolytic degradation [8]. Ctr1 endocytosis is induced when cells are exposed to 0.1 to 1 µM copper, whereas Ctr1 degradation occurs in response to copper concentrations of 10 µM or more. Cells defective in endocytosis, due to mutations in the general endocytosis system, still undergo copper-stimulated Ctr1 proteolysis, suggesting that degradation occurs at the plasma membrane via a non-classical degradative pathway [8]. The observation that Ctr1 undergoes copper-induced endocytosis was confirmed by a second study [15]. The second investigation concluded that Ctr1 is ubiquitinylated in a copper- and Rsp5-dependent manner. Consequently, the copper-dependent recognition of Ctr1 by the Rsp5 ubiquitin ligase, which is required for ubiquitylation and degradation of a wide variety of transmembrane proteins, is followed by the delivery of Ctr1 to the lumen of the vacuole, and then by its subsequent degradation by vacuolar proteases [15]. Unlike these first two reports, a third study indicated that the Ctr1 transporter is neither regulated at the level of subcellular localization nor endocytosed by copper [16]. Instead, this study proposed a model in which excess copper induces conformational changes in the cytosolic C-terminal tail of Ctr1, inhibiting the passage of copper across the plasma membrane [16].

In mammals, Ctr transporters represent an important point of regulation with which to adjust the intracellular concentration of copper as a function of environmental copper availability [1], [3]. However, as in the case of *S. cerevisiae* Ctr1, the proposed mechanisms of post-translational regulation of CTR1 in human (hCTR1) and mice (mCTR1) differed between studies [9]–[11], [17]. In human embryonic kidney (HEK293) cells, it has been reported that high copper levels trigger endocytosis and degradation of hCTR1 [9], [18]. Other experiments have revealed that only a fraction of hCTR1 is internalized in response to copper, and that hCTR1 internalization is accompanied by its recycling to the plasma membrane when the cells undergo a shift from high to low copper levels [17]. Other imaging studies have shown that high copper levels have no effect on hCTR1 localization at the cell surface [10], [11]. Conversely, other reports have localized endogenous hCTR1 and mCTR1 both at the plasma membrane and in association with as-yet undefined intracellular vesicles [10], [19].

Copper is transported by a dual partner complex formed by two integral membrane proteins encoded by the *ctr4^+^* and *ctr5^+^* genes in *Schizosaccharomyces pombe* [20]–[22]. A clear interdependence between Ctr4 and Ctr5 has been established, since the trafficking of either protein to the cell surface requires the concomitant trafficking of the other [21], [22]. Analysis of fission yeast cells expressing Ctr4 and Ctr5 proteins in which their N-terminal regions have been mutated, revealed that at least one N-terminal region provided by either protein suffices for copper transport [22]. Transcription of the *ctr4^+^* and *ctr5^+^* genes is regulated by copper availability via the copper sensing transcription factor 1 (Cuf1) [21], [23]. These genes are induced under conditions of copper starvation, and are turned off under conditions of copper repletion. Because studies in *S. cerevisiae* and mammalian cell lines have suggested that Ctr proteins appeared to be post-translationally regulated, we examined whether the *S. pombe* heteromeric Ctr4–Ctr5 transport system was subjected to a form of post-translational regulation in response to changes in copper levels. To begin to address this question, we have developed a bimolecular fluorescence complementation (BiFC) assay [24], [25] for use in fission yeast, allowing us to study the Ctr4–Ctr5 complex in living cells as a function of copper availability. Using this method, we determined that the Ctr4 and Ctr5 proteins at the plasma membrane underwent internalization from the cell surface after copper addition. This copper-mediated internalization of the Ctr4–Ctr5 complex was reversible, and was not dependent on *de novo* protein synthesis. Collectively, our results strongly suggest that copper-dependent internalization and recycling may be an important mechanism with respect to fine tuning of the number of Ctr4–Ctr5 molecules present at the plasma membrane in fission yeast.

## Results

### BiFC system for visualizing the Ctr4–Ctr5 complex in *S. pombe in vivo*

**Figure 1:**
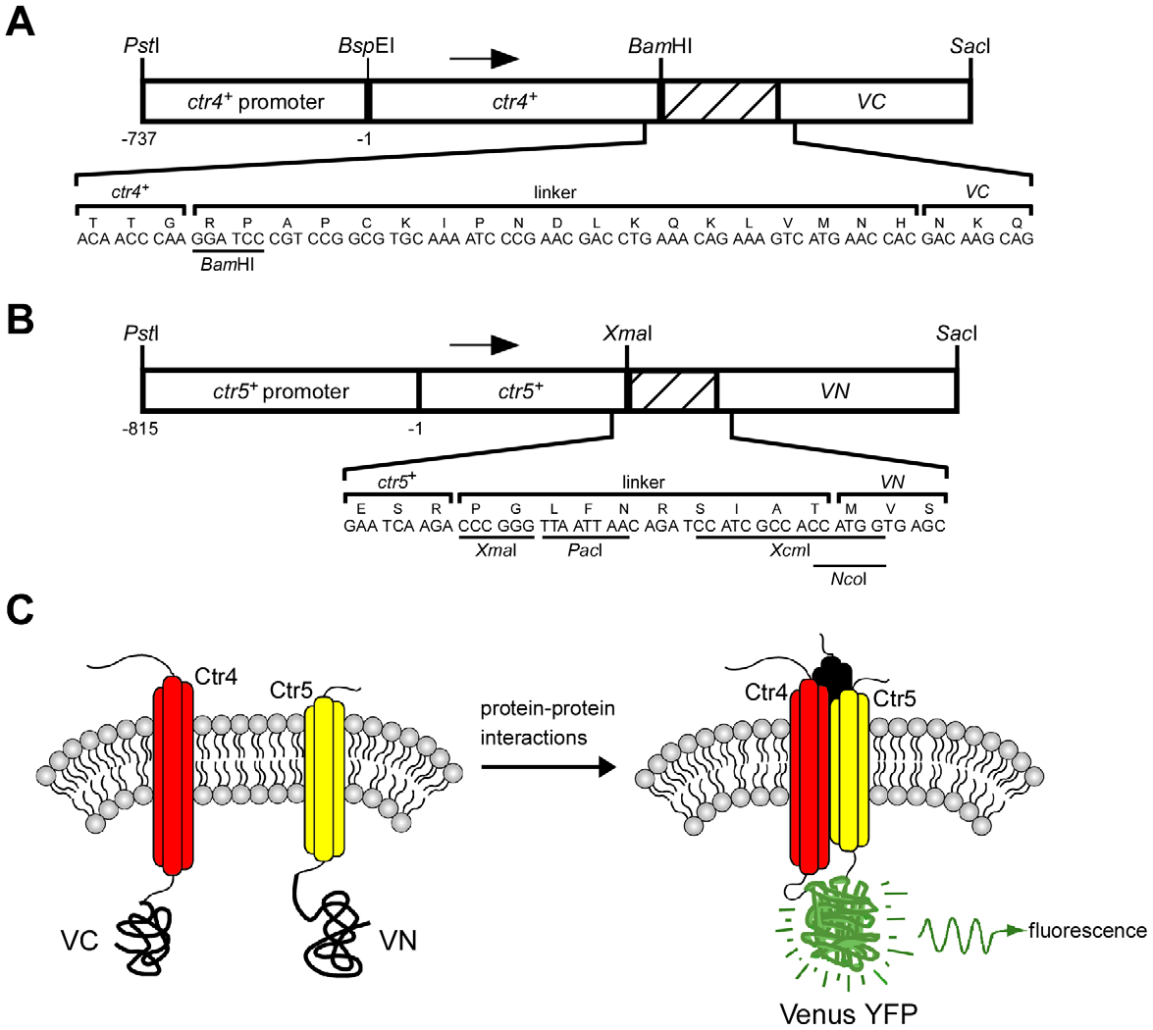
Design of the BiFC system used in *S. pombe*. *A*, Schematic representation of the *ctr4^+^-VC* fusion gene including a linker region (*hatched box*) that was inserted in-frame of and between the *ctr4^+^* allele and the nonfluorescent C-terminal (VC) coding fragment of Venus. The *ctr4^+^-VC* fusion allele is under the control of its native promoter. The numbers refer to the position of the nucleotides relative to the translational initiator codon of *ctr4^+^*. *B*, The VN DNA fragment was fused downstream of and in-frame with the *ctr5^+^* allele, which itself is under the control of its endogenous promoter. The *hatched box* represents a DNA sequence derived from a linker region. The amino acid sequence of each linker (57 base pairs (bps) for *ctr4^+^-VC* and 30 bps for *ctr5^+^-VN*) is depicted using the single-letter amino acid code. *C*, Schematic representation of Ctr4-VC and Ctr5-VN fusion proteins in a membrane. Transmembrane proteins Ctr4-VC and Ctr5-VN are represented by round-shaped red and yellow cylinders, respectively. Each cylinder represents a transmembrane domain. A third putative transmembrane protein partner (represented in dark) corresponds to a putative monomer of Ctr4 or Ctr5. Ctr4-VC and Ctr5-VN close association reconstitutes a functional fluorophore.

**Figure 2:**
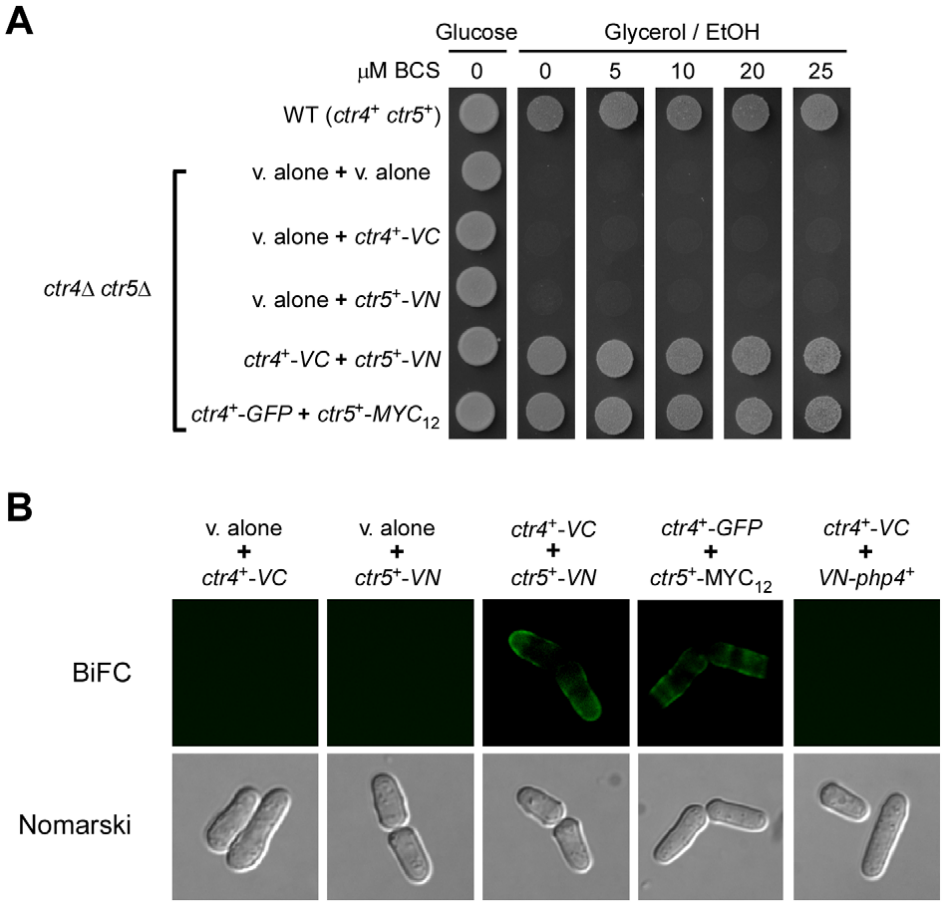
Co-expression of Ctr4-VC and Ctr5-VN functionally complements the respiratory deficiency of a *ctr4Δ ctr5Δ* mutant and produces a BiFC signal at the plasma membrane. *A*, *S. pombe* cells harboring a *ctr4Δ ctr5Δ* double deletion were transformed with empty vectors (v. alone), a vector alone (v. alone) and *ctr4^+^-VC*, a vector alone and *ctr5^+^-VN*, *ctr4^+^-VC* and *ctr5^+^-VN*, or *ctr4^+^-GFP* and *ctr5^+^-MYC_12_*. Cultures were spotted onto YES media containing glucose or glycerol/ethanol (EtOH) and BCS (0, 5, 10, 20 and 25 µM). WT, isogenic wild-type (WT) strain FY435 (*ctr4^+^ ctr5^+^*). *B*, BiFC signal of Ctr4-VC and Ctr5-VN fusion proteins at the plasma membrane. Yeast cells disrupted for *ctr4^+^* and *ctr5^+^* that expressed individually, or in combination, the indicated tagged genes were grown in EMM medium containing BCS (100 µM) and analyzed by fluorescence microscopy. As a positive control, co-expression of the Ctr4-GFP and Ctr5-Myc12 fusion proteins allowed Ctr4-GFP detection in the plasma membrane. As an additional proof of the specificity of the BiFC, no Venus-associated fluorescence was detected when the unrelated *ctr4^+^-VC* and *VN-php4^+^* fusion alleles were co-expressed together. Cell morphology was examined through Nomarski optics.

Two integral membrane proteins, Ctr4 and Ctr5, form a copper transporting complex at the cell surface of *S. pombe* that is required for growth under low copper conditions [21], [22]. Previous studies have revealed that cells harboring a deletion of either *ctr4^+^* or *ctr5^+^* are defective in high affinity copper uptake [21]. Consistently, these mutant cells exhibit phenotypes linked with copper deficiency, including the inability to grow on respiratory carbon sources and an impaired copper amine oxidase activity [21], [22], [26]. To begin to characterize this heteroprotein complex *in vivo* as a function of copper availability, a BiFC approach was used in fission yeast. To establish this method, the Venus protein was used [27]. Venus is an improved version of the yellow fluorescent protein (YFP) that contains amino acid substitutions, F46L/F64L/M153T/V163A/S175G, which greatly accelerates its folding and maturation [27]. Thus, fragments of Venus that fold rapidly form BiFC complexes more efficiently than fragments of YFP that fold slowly. Furthermore, Venus is the brightest fluorescent protein that has been identified, allowing the highest intensity of BiFC complex fluorescence [27]. Venus carboxyl-terminal fragment (VC) and Venus amino-terminal fragment (VN) were fused to the C-terminal ends of Ctr4 and Ctr5, respectively. Using either the pBP*ade6^+^* [22] or pJK148 [28] plasmid as a backbone, the *ctr4^+^* locus, which includes its own promoter, was inserted downstream of and in-frame with the VC coding fragment of Venus (Fig. 1). A flexible linker region was inserted between the DNA fragments encoding Ctr4 and VC, and was derived from pFA6a-VC-kanMX6 [25] (Fig. 1A). Similarly, the *ctr5^+^* gene, under the control of its own promoter, was fused to the coding region of VN using a flexible linker sequence derived from pFA6a-VN-kanMX6 [25] (Fig. 1B). The working hypothesis was that the association between the two nonfluorescent VC-tagged Ctr4 and VN-tagged Ctr5 proteins should trigger the assembly of a functional Venus fluorophore, thereby allowing the detection of a BiFC signal by fluorescence microscopy (Fig. 1C). Plasmids expressing the VC-tagged Ctr4 and VN-tagged Ctr5 proteins were co-transformed into a *ctr4Δ ctr5Δ* strain and the cells were analyzed for respiratory competency as compared to cells transformed with functional Ctr4-GFP (green fluorescent protein) and Ctr5-Myc_12_ proteins [22]. As we have previously shown, the GFP-tagged Ctr4 and Myc_12_-tagged Ctr5 proteins used in this study (as positive controls) functionally complement the respiratory deficiency of a *ctr4Δ ctr5Δ* strain in a manner indistinguishable from the *ctr4^+^* and *ctr5^+^* wild-type alleles [22]. When Ctr4-VN and Ctr5-VC were co-expressed in *ctr4Δ ctr5Δ* cells, they functionally complemented the respiratory deficiency of the double mutant strain at the same level as did the Ctr4-GFP and Ctr5-Myc_12_ fusion proteins (Fig. 2A). As expected, the wild-type parental strain (used as a control), which harbored chromosomal copies of the *ctr4^+^* and *ctr5^+^* genes, grew effectively on respiratory carbon sources due to the ability of the Ctr4–Ctr5 complex to transport copper to the copper-requiring cytochrome *c* oxidase (Fig. 2A). In contrast, a *ctr4Δ ctr5Δ* double mutant strain that was co-transformed with two empty vectors, an empty vector and *ctr4^+^-VC*, or an empty vector and *ctr5^+^-VN*, failed to grow on respiratory carbon sources. This latter phenotype was presumably due to the inability of the mutant cells to provide copper to the cytochrome *c* oxidase [29].

When Ctr4-VC and Ctr5-VN were co-expressed in *ctr4Δ ctr5Δ* cells under conditions of copper starvation, they generated a BiFC signal at the plasma membrane in a manner similar to that of the full-length version of Ctr4-GFP (that was co-transformed with Ctr5-Myc_12_) (Fig. 2B). The specificity of the BiFC signal was validated by the lack of a fluorescence signal when either the Ctr4-VC or Ctr5-VN fusion protein was expressed in the absence of either Ctr5-VN or Ctr4-VC, respectively (Fig. 2B). Furthermore, no BiFC signal was detected in *ctr4Δ ctr5Δ* cells co-expressing two unrelated proteins harboring the N- and C-terminal fragments of Venus, such as VN-Php4 [30] and Ctr4-VC (Fig. 2B). These results show that, consistent with their role in copper uptake, the Ctr4-VC and Ctr5-VN fusion proteins generate a BiFC signal at the plasma membrane in living cells.

### Requirement of two Ctr4 monomers for localization at the plasma membrane

**Figure 3:**
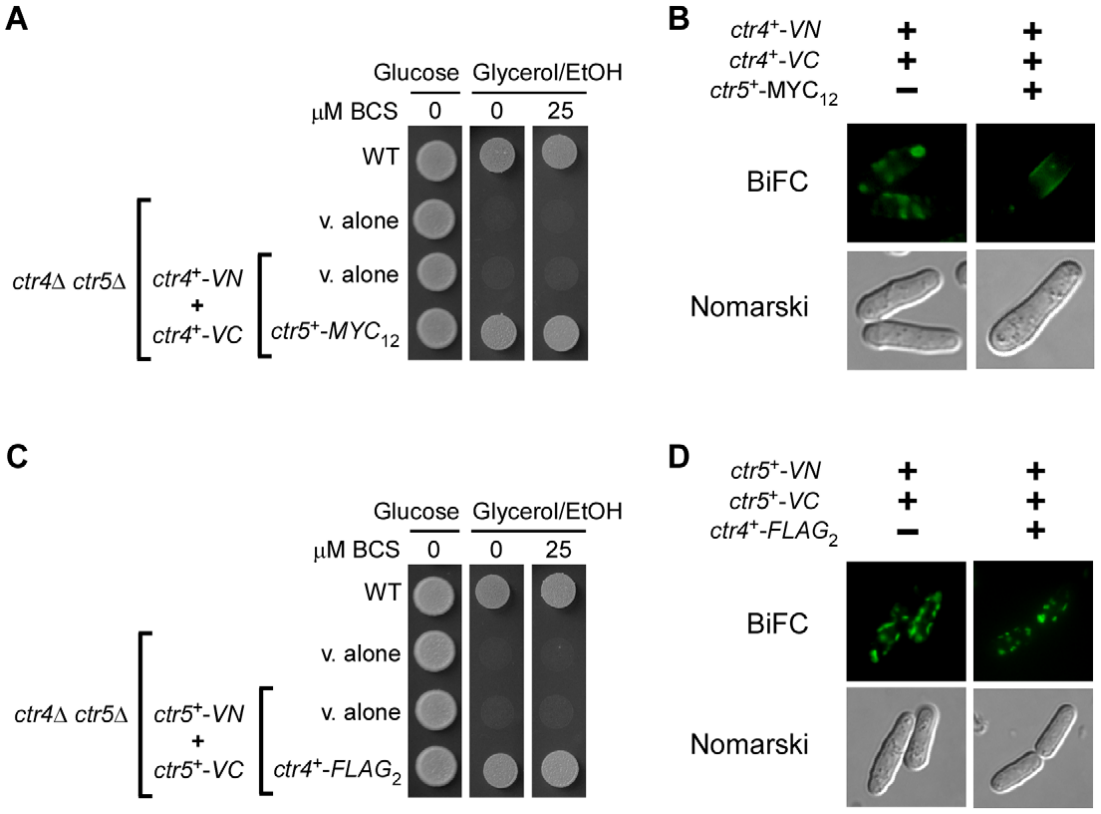
Cells co-expressing Ctr4-VN and Ctr4-VC in the presence of Ctr5-Myc_12_ display a fluorescent signal at the plasma membrane, whereas Ctr5-VN and Ctr5-VC co-expressed in the presence of Ctr4-Flag_2_ fail to interact at the plasma membrane. *A*, The strain JSY22 (*ctr4Δ ctr5Δ*) was co-transformed with vectors alone (v. alone), integrative plasmids co-expressing only *ctr4^+^-VN* and *ctr4^+^-VC*, or *ctr4^+^-VN* and *ctr4^+^-VC* in the presence of *ctr5^+^-MYC_12_*. Cells were grown either for 5 days on YES medium in the presence of glucose or, for 9 days, on YES medium in the presence of glycerol/ethanol (EtOH) containing or not BCS (25 µM). WT, parental wild-type strain FY435. *B*, *ctr4Δ ctr5Δ* cells co-expressing the indicated fusion alleles were grown to an *A_600_* of 0.5. At this optical density, BCS (100 µM) was added and the treated cultures were incubated for 3 h at 30°C, and then visualized for BiFC by fluorescence microscopy. The cells were also examined by Nomarski microscopy for cell morphology. *C*, JSY22 (*ctr4Δ ctr5Δ*) cells were co-transformed with vectors alone, or with *ctr5^+^-VN* and *ctr5^+^-VC* in the absence (v. alone) or the presence of *ctr4^+^-FLAG_2_*. Growth was tested on both fermentable (glucose) and non-fermentable (glycerol/ethanol) media that were either supplemented, or not with BCS (25 µM). *D*, BiFC analysis of Ctr5-VN + Ctr5-VC and Ctr5-VN + Ctr5-VC + Ctr4-FLAG_2_ in *ctr4Δ ctr5Δ* cells. Representative fluorescence images of BiFC are shown. Nomarski microscopy was used to determine cell morphology.

Genetic trans-complementation, biochemical and structural projection experiments have demonstrated that Ctr transporters need to trimerize in order to function in copper uptake [6], [7], [14], [31]. Given the fact that in *S. pombe*, both Ctr4 and Ctr5 proteins are physically associated and interdependent for their localization at the plasma membrane, we sought to determine whether Ctr4-VN and Ctr4-VC co-localized at the plasma membrane in the presence of Ctr5-Myc_12_. Co-expression of both *ctr4^+^-VN* and *ctr4^+^-VC* in cells expressing *ctr5^+^-Myc_12_* allowed *ctr4Δ ctr5Δ* cells to grow on a glycerol/ethanol medium (Fig. 3A). With this combination of fusion proteins, the BiFC signal was localized at the plasma membrane (Fig. 3B). We determined that *ctr4Δ ctr5Δ* cells co-expressing only *ctr4^+^-VN* and *ctr4^+^-VC* were unable to grow on glycerol/ethanol medium (Fig. 3A). Analysis of the localization of the Ctr4-VN-Ctr4-VC complex by fluorescence microscopy revealed that the BiFC signal was mainly detected in the cytosol of the cells, without distinct fluorescence visible at the periphery of the cells (Fig. 3B). Accordingly, these results corroborated previous findings by our group and others that both Ctr4 and Ctr5 must be co-expressed to form a copper-transporting complex at the plasma membrane [21], [22]. As previously shown, Ctr4 is mislocalized within the secretory pathway in the absence of Ctr5 [21], [22]. Based on this result, we tested the co-expression of *ctr5^+^-VN* and *ctr5^+^-VC* in cells expressing *ctr4^+^-Flag_2_*. Although *ctr4Δ ctr5Δ* cells expressing these fusion alleles were able to grow on glycerol/ethanol, no BiFC signal was detected at the plasma membrane (Fig. 3, C and D). Instead, BiFC was only detected as a punctual fluorescence within the cells. These results strongly suggest that only heteromeric protein complexes including two Ctr4-Flag_2_ monomers with a single Ctr5-VN, or two Ctr4-Flag_2_ monomers with a single Ctr5-VC, were competent to confer growth on respiratory carbon sources. Collectively, these data support the notion that two Ctr4 monomers with a combination of one Ctr5 monomer are required for localization of the heteromeric Ctr complex to the plasma membrane and its proper functioning in copper transport in *S. pombe*.

### Copper-induced internalization of the Ctr4–Ctr5 complex

**Figure 4:**
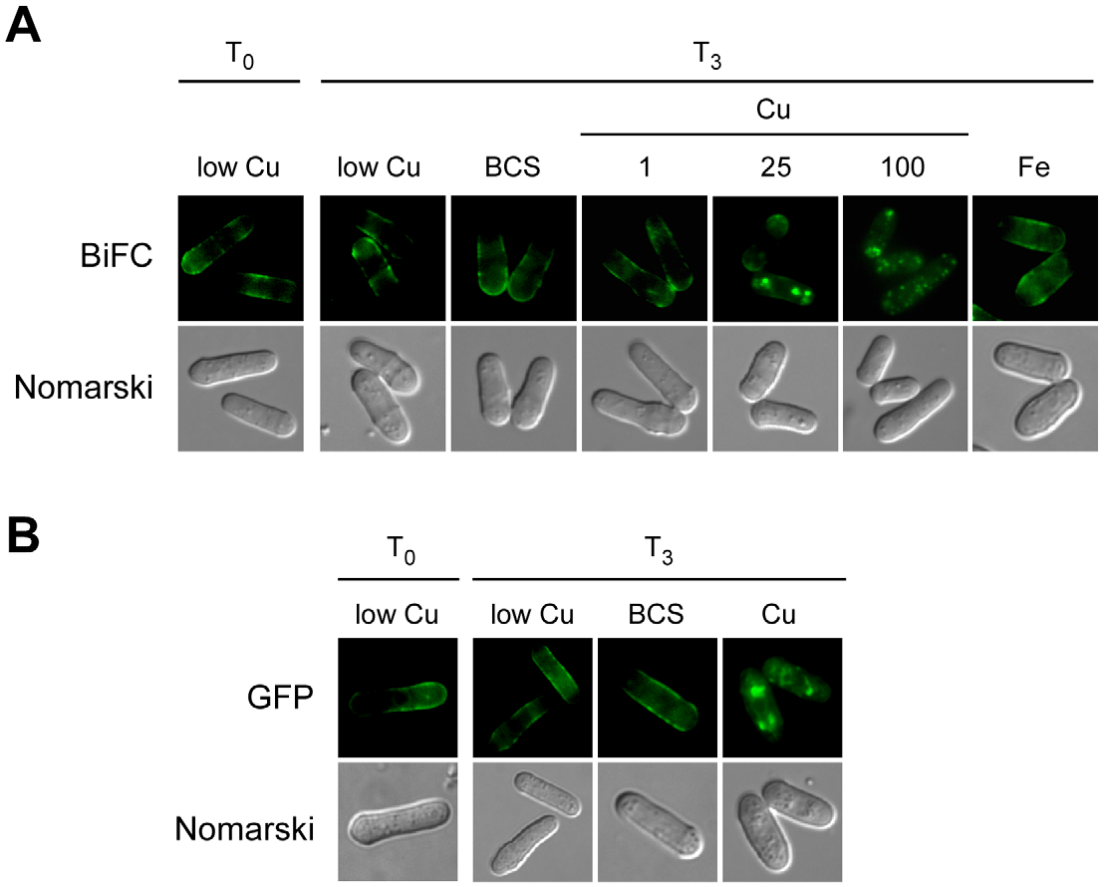
Copper-dependent internalization of the Ctr4-Ctr5 complex. *A*, Cells harboring a *ctr4Δ ctr5Δ* double deletion were co-transformed with the *ctr4^+^-VC* and *ctr5^+^-VN* alleles. Co-transformed cells were grown in EMM containing 160 nM of copper (low Cu) to an *A_600_* of 0.5 (T_0_), and then were left untreated (low Cu), or were treated with BCS (100 µM), CuSO_4_ (1, 25, and 100 µM) (Cu) or FeCl_3_ (100 µM) (Fe). After being incubated for 3 h, the cells were visualized by fluorescence microscopy (BiFC). The cells were also examined by Nomarski microscopy for cell morphology. *B*, *ctr4Δ ctr5Δ* cells co-expressing the Ctr4-GFP and Ctr5-MYC_12_ fusion proteins were grown to mid-logarithmic phase in EMM copper-poor (160 nM) media (low Cu). Cells were then incubated in the absence (low Cu) or the presence of CuSO_4_ (100 µM) or BCS (100 µM). After a 3 h treatment, the full-length Ctr4-GFP protein was viewed by direct fluorescence microscopy (GFP). The corresponding Nomarski images are also shown for each GFP panel.

To assess the effect of copper on the localization of the Ctr4–Ctr5 heteromeric plasma membrane complex, *ctr4Δ ctr5Δ* cells co-transformed with plasmids expressing the Ctr4-VC and Ctr5-VN proteins were first grown to mid-logarithmic phase under low copper conditions, so as to ensure a plasma membrane localization of the Ctr4-VC-Ctr5-VN complex (Fig. 4A, T_0_). The cells were then washed and resuspended in the same copper-limited medium (160 nM), which was left unsupplemented or supplemented with the copper chelator bathocuproinedisulfonic acid (BCS; 100 µM), FeCl_3_ (100 µM) or CuSO_4_ at various concentrations (1, 25 or 100 µM), for 3 h. Results showed that when the cells were exposed to 25 and 100 µM CuSO_4_, the Ctr4-VC-Ctr5-VN protein complex accumulated intracellularly in a copper concentration-dependent manner (Fig. 4A). The BiFC signal exhibited copper-dependent internalization from the cell surface, with the progressive appearance of vesicular fluorescence. In contrast, following treatment with FeCl_3_ (100 µM) or BCS (100 µM) for 3 h, the Ctr4-VC-Ctr5-VN complex remained at the plasma membrane (Fig. 4A). To ascertain whether, like Ctr4-VC-Ctr5-VN, Ctr4-GFP underwent a copper-dependent internalization when it was co-expressed with Ctr5-Myc_12_ in the presence of high copper concentrations, the above-mentioned cell growth protocol was repeated using *ctr4Δ ctr5Δ* cells expressing Ctr4 and Ctr5 proteins that were tagged with GFP and Myc_12_, respectively. Results revealed that, as visualized using the BiFC assay with Ctr4-VC-Ctr5-VN, Ctr4-GFP exhibited copper-induced internalization from the cell surface in response to exposure to 100 µM CuSO_4_ (Fig. 4B). In contrast, when cells co-transformed with plasmids expressing the Ctr4-GFP and Ctr5-Myc_12_ proteins were left untreated or were treated with BCS (100 µM), Ctr4-GFP remained at the cell surface (Fig. 4B). The results indicate that upon exposure of *S. pombe* to high copper conditions, Ctr4-VC-Ctr5-VN heteromeric complexes underwent internalization from the cell surface, producing BiFC signals that had the appearance of cytoplasmic punctuate structures within the cell.

### Copper-induced internalization of pre-existing heteromeric Ctr4–Ctr5 molecules

**Figure 5:**
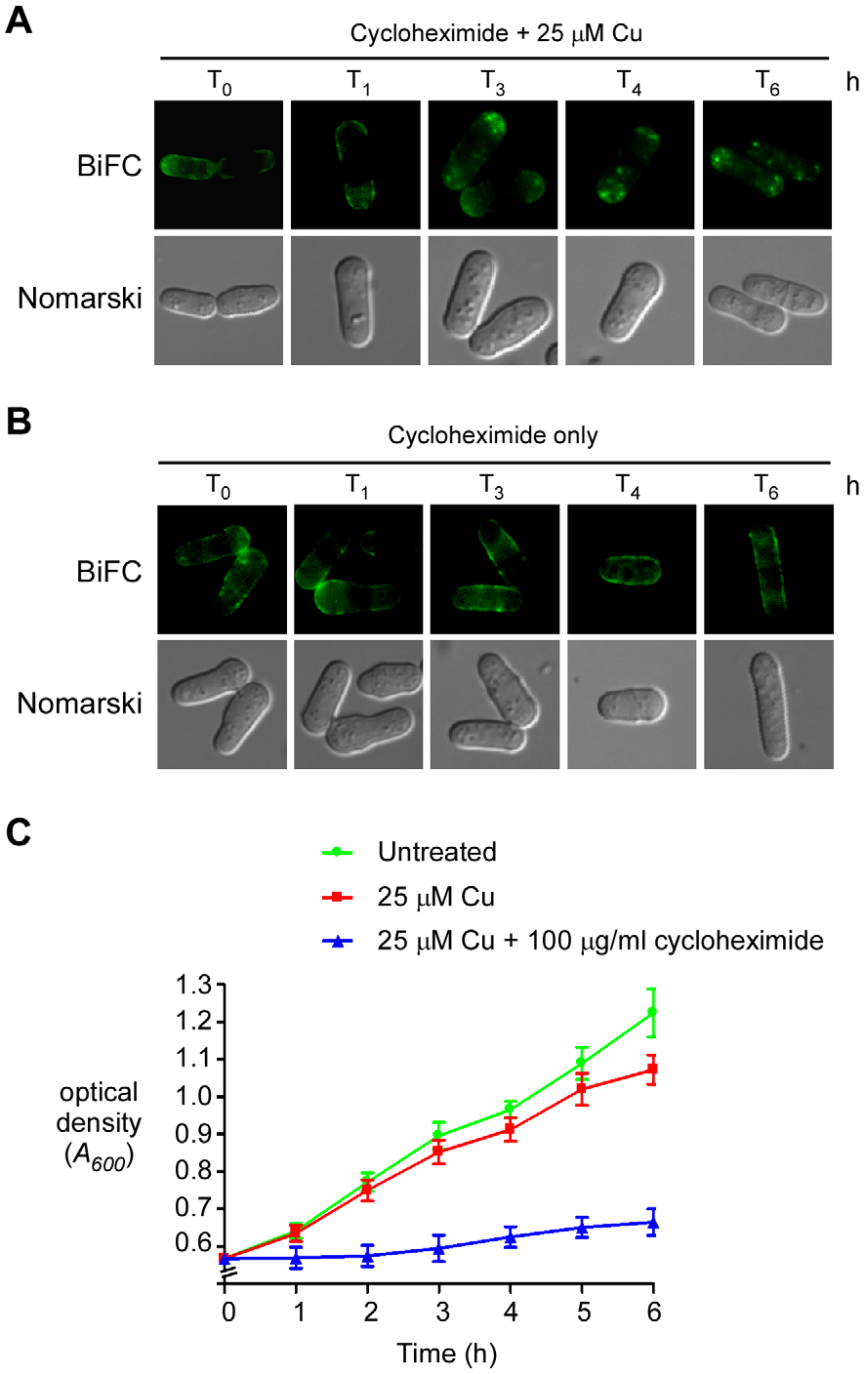
Preexisting Ctr4-Ctr5 complexes undergo internalization in response to high concentrations of copper. *A*, A double-disruption strain (*ctr4Δ ctr5Δ*) was co-transformed with the *ctr4^+^-VC* and *ctr5^+^-VN* alleles. A mid-logarithmic phase culture (*A_600_* of 0.5) was treated with cycloheximide (100 µg/ml) for 30 min so as to inhibit protein synthesis, followed by treatment with CuSO_4_ (25 µM) for the indicated times. To ensure the complete inhibition of protein synthesis, the cycloheximide treatment was repeated after 3 h of exposure of the cells to copper. The cells were then analyzed by fluorescence microscopy (BiFC). Cell morphology was examined using Nomarski optics. *B*, Cells were cultured as described for panel A, except that they were treated with cycloheximide only, without exposure of the cells to CuSO_4_. *C*, Growth of cells was assessed in liquid cultures in the absence (untreated) or the presence of CuSO_4_ (25 µM), or a combination of CuSO_4_ and cycloheximide (100 µg/ml). All cultures were restarted at an *A_600_* of ∼0.5. Data are the average of triplicate samples from three independent cultures. Error bars indicate the average ± standard deviation.

Cells expressing *ctr4^+^-VC* and *ctr5^+^-VN* were treated with cycloheximide to ensure that the cytoplasmic punctuate BiFC signal was due to the copper-dependent internalization of pre-existing Ctr4-VC-Ctr5-VN complexes from the cell surface and not to the effect of copper on newly synthesized Ctr4-VC-Ctr5-VN complexes arising from a pool of stable mRNA. Cytoplasmic translation was inhibited by addition of cycloheximide (100 µg/ml) for 30 min, followed by treatment with CuSO_4_ (25 µM) for 0, 1, 3, 4 and 6 h. Under these conditions, the Ctr4-VC-Ctr5-VN complexes that were present at the cell surface were internalized in response to copper treatment (Fig. 5A). This copper-stimulated internalization was detectable after 3 h, and remained observable for at least 6 h. The failure of cycloheximide to influence this process indicated that copper-induced intracellular trafficking of Ctr4-Ctr5 complexes was not dependent on the *de novo* synthesis of proteins. Furthermore, Ctr4-VC-Ctr5-VN complexes were stable at the cell surface when cells were grown under low copper concentrations and in the presence of cycloheximide, indicating that the Ctr4–Ctr5 complex internalization was specific to copper treatment (Fig. 5B). We checked that the cycloheximide treatment had an inhibitory growth effect on cells harboring Ctr4-VC-Ctr5-VN complexes (Fig. 5C). Although cells grown in cycloheximide-free medium containing copper (25 µM) showed 23% inhibition of growth after 6 h of incubation, as compared to cells that were left untreated (neither with copper nor cycloheximide), cells that had been treated with cycloheximide in the presence of copper exhibited a much more pronounced growth inhibition (85%) at this time point (6 h) (Fig. 5C). Because the growth rate of cycloheximide-treated cells was significantly inhibited as compared to control cells, we concluded that *de novo* protein synthesis was blocked by cycloheximide in an effective manner. Taken together, these results indicate that the localization of Ctr4–Ctr5 complexes at the plasma membrane is regulated in response to copper through the relocalization of the heteroprotein complex from the cell surface to the cytoplasm.

### Copper-dependent recycling of Ctr4–Ctr5 proteins

**Figure 6:**
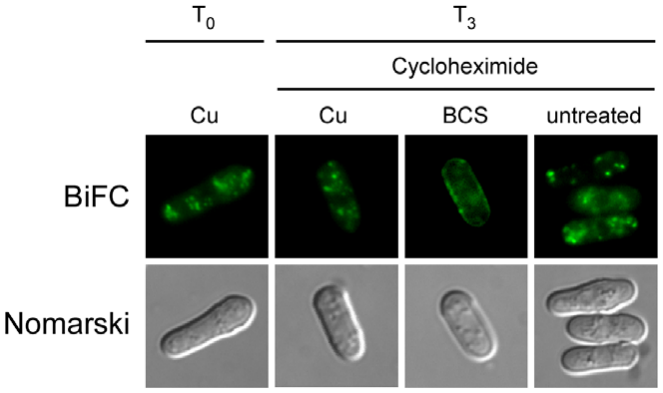
Ctr4-Ctr5 complexes recycle during a shift of high to low copper concentrations. *ctr4Δ ctr5Δ* cells containing the tagged *ctr4^+^-VC* and *ctr5^+^-VN* coding sequences were grown in EMM containing CuSO_4_ (160 nM) for 16 h. The culture was then diluted (1×10^7^ cells/ml) and incubated in the presence of CuSO_4_ (25 µM) for 4 h, at which time (T_0_) the cells were washed twice and incubated in the presence of cycloheximide (100 µg/ml) for 30 min. At this point, the culture was divided into three treatment groups. A first group was left untreated (−), while the two other were treated with 25 µM CuSO_4_ (Cu) or 250 µM BCS, respectively. Nomarski microscopy was used to determine cell morphology.

The above results revealed that, in response to high concentrations of copper, Ctr4–Ctr5 complexes were internalized in the cytoplasm. In order to test whether Ctr4-VC-Ctr5-VN complexes recycled back to the plasma membrane under conditions of low copper, cells were grown in the presence of 25 µM copper for 4 h, after which step (Fig. 6, T_0_), they were examined as a function of copper availability. Following the 4 h incubation in the presence of exogenous copper, the cells were harvested and then resuspended in basal medium in the presence of cycloheximide (100 µg/ml) for 30 min. At this time point, the localization of the BiFC signal was unchanged and similar to that of the zero time point (T_0_, data not shown). Following the 30 min exposure to cycloheximide, the cells were divided into three groups. A first group was left untreated, while the two other groups were incubated in the presence of exogenous copper (25 µM) or BCS (250 µM). When cells were starved for copper in the presence of BCS, the BiFC signal returned to the plasma membrane (Fig. 6). The possible implication of newly synthesized Ctr4-VC-Ctr5-VN complexes was eliminated by pre-treatment with cycloheximide. In contrast, the incubation performed in the presence of copper (25 µM) resulted in the detection of the BiFC signal in cytoplasmic punctuated structures as observed at the onset of the observations (T_0_, Fig. 6). Similarly, when the cells were re-incubated in basal medium (untreated), there was an absence of copper starvation-dependent recycling of Ctr4-VC-Ctr5-VN to the plasma membrane (Fig. 6). Under this latter experimental condition, the BiFC signal remained localized to the cytosol and exhibited a punctuated distribution. The BiFC data strongly suggest a model in which the elevation of copper levels results in a copper-stimulated internalization of the Ctr4–Ctr5 copper transport complex. In contrast, the Ctr4–Ctr5 molecules that had been internalized in response to excess copper undergo recycling back to the plasma membrane in response to a transition from high to copper-limiting conditions.

## Discussion

One purpose of the experiments described in this work was to develop and utilize BiFC to study protein-protein interactions in live fission yeast cells. The Ctr4–Ctr5 association was selected as a proof-of-concept model, since this interaction has previously been established in cells grown under conditions of copper starvation [21], [22]. Under copper-limiting conditions, studies have shown that the simultaneous biosynthesis of Ctr4 and Ctr5 is required for their appropriate targeting to the cell surface as a heteromeric complex, as well as for their ability to mediate high affinity copper uptake [21], [22]. In the present study, using the BiFC method to investigate protein-protein interactions and localization within live cells, we showed that the Ctr4–Ctr5 heterocomplex was regulated post-translationally since copper induced its internalization. However, copper-dependent transcriptional inhibition of *ctr4^+^* and *ctr5^+^* gene expression is more sensitive than post-translational regulation of the Ctr4–Ctr5 complex. Copper, at concentrations as low as 1 µM, can repress the transcription of *ctr4^+^* and *ctr5^+^* ([23], [32] and data not shown), whereas the same concentration had no apparent effect on the localization of the heteromeric Ctr4–Ctr5 complex at the cell surface (Fig. 4). The data we obtained represent the more long term effects of elevated copper on the Ctr4–Ctr5 transporting complex. Indeed, the copper-dependent post-transcriptional internalization of the heteromeric Ctr4–Ctr5 complex was observed only 3 h after copper addition (Fig. 5). This suggests that the copper-induced internalization of the Ctr4–Ctr5 heterocomplex is not the primary mechanism for inhibiting the transport of copper. In fact, copper transport appears to be uncoupled from target gene regulation. In this context, it has been shown that transcriptional regulation is more sensitive than post-translational regulation for both high affinity iron and zinc transport systems in *S. cerevisiae* [33]–[35]. For instance, iron, at levels as low as 1 to 10 µM, can repress transcription of the *S. cerevisiae FET3* gene by >90%, whereas the same concentrations only had minimal effects on protein steady-state levels.

Structural and biochemical studies have revealed that membrane proteins of the Ctr family exhibit a trimeric organization, allowing the formation of a translocation path that includes nine transmembrane domains [36]. In contrast to Ctr1 and Ctr3 from *S. cerevisiae* [14], [16], [37], and human CTR1 [38], that self-trimerize in the plasma membrane, Ctr4 and Ctr5 from *S. pombe* are interdependent for co-localization at the cell surface and participation in copper transport [21], [22]. We co-expressed *ctr4^+^-VN* and *ctr4^+^-VC* in cells expressing *ctr5^+^-Myc_12_* to better characterize the nature of this copper-transporting complex in living cells. Because this was the only pair of interacting proteins that allowed their detection at the plasma membrane when co-expressed with Ctr5, we proposed a model in which the formation of a heteromeric Ctr4–Ctr5 complex required the presence of at least two Ctr4 monomers with a combination of one Ctr5 monomer, resulting in a 2/1 (Ctr4/Ctr5) subunit ratio. However, our data did not allow us to conclusively establish whether the Ctr4–Ctr5 copper-transporting complex assembled strictly as a trimer or whether it adopted a higher-ordered quaternary structure at the cell surface. Based on a structural model of Ctr transporters [7], [36], it has been observed that Ctr4 possesses residues at positions 236 (Phe) and 237 (Leu) in the third transmembrane domain that may hinder the formation of a stable homo-multimeric complex. Consequently, it has been proposed that Ctr5 may ensure stable assembly of the copper transport complex. Because Ctr5 was originally identified based on its ability to alleviate a block in Ctr4 sorting to the plasma membrane, it is quite likely that the Ctr5 protein is required for Ctr4 hetero-oligomerization and to exit from the secretory pathway, where the heterocomplex can mediate copper uptake into the cell. Studies are currently ongoing in our laboratory to test this possibility.

One group has reported that the presence of excess copper at a concentration of 10 µM rapidly induced the degradation of Ctr1 at the plasma membrane (half-life<30 min) in *S. cerevisiae* [8]. It was suggested that the copper-dependent degradation of Ctr1 occured in the absence of endocytosis. Another group has shown that at higher concentrations of copper (50 µM), Ctr1 was partially internalized by endocytosis, with a small proportion of the internalized protein being degraded 2 h after the addition of copper [15]. Recently, an additional group has concluded that within 30 min of exposure to excess copper (100 µM), plasma membrane-associated Ctr1 levels were not significantly affected [16]. These authors proposed a mechanism by which conformational changes in the C-terminal cytosolic tail of Ctr1 lead to inhibition of the Ctr1-mediated copper uptake [16]. Consistent with the proposed model, cells expressing altered forms of Ctr1 in which the C-terminal cytosolic region was either truncated or mutated were very sensitive to excess copper [16]. However, a potential relocalization of Ctr1 following long periods of cell exposure to copper (>30 min) has not yet been reported. Nonetheless, site-directed mutagenesis has identified amino acid residues within the Ctr1 C-terminal cytosolic tail that are important for blocking the Ctr1-dependent copper uptake in response to excess copper. Among these residues, five Cys located at positions 304, 310, 312, 363 and 365, as well as a dileucine motif (Leu-397 and Leu-398), have been shown as being required for the inhibition of copper translocation across the plasma membrane. Although *S. pombe* Ctr4 and Ctr5 function in high affinity copper transport at the cell surface, their C-terminal cytosolic tails are different in both length and amino acid sequence composition as compared to that of *S. cerevisiae* Ctr1. The C-terminal tail of Ctr1 contains 125 amino acid residues, whereas the C-terminal cytosolic regions of Ctr4 and Ctr5 are shorter, encompassing only 39 and 16 amino acid residues, respectively. Ctr4 and Ctr5 do not contain a dileucine motif within their C-termini. Furthermore, the C-terminal cytosolic region of Ctr4 lacks Cys residues, while that of Ctr5 contains a single Cys-Cys motif located at positions 169 and 170. Because of the important variations in both length and composition of residues between the C-terminus of Ctr1 and the C-terminal cytosolic tails of Ctr4 and Ctr5, it can be argued that some aspects of transporter regulation differ between different family members. Analogous to recent results with hCTR1 [17], the Ctr4–Ctr5 complex was observed to undergo internalization and intracellular accumulation in response to elevated environmental copper levels. Furthermore, as observed in the case of hCTR1 [17], the heteromeric Ctr4–Ctr5 complex is recycled back to the plasma membrane following removal of bioavailable exogenous copper.

It has been shown that either the deletion of the C-terminal cytosolic tail of *S. cerevisiae* Ctr1 or the insertion of an epitope tag within the same region of Ctr1 resulted in a structural perturbation that inhibited its function in preventing excess copper uptake [16]. Consequently, cells expressing these mutant alleles exhibited a copper-sensitive growth phenotype. Based on these observations, we determined whether the VN or VC epitope-tagged versions of the Ctr4 or Ctr5 proteins confered sensitivity to copper when expressed in *ctr4Δ ctr5Δ* cells. We observed that cells constitutively co-expressing *ctr4^+^-VN* or *ctr4^+^-VC* in combination with *ctr5^+^-VN* or *ctr5^+^-VC* did not exhibit copper-sensitive growth phenotype (data not shown). Indeed, when the tagged *ctr4^+^* and *ctr5^+^* alleles were co-expressed in *ctr4Δ ctr5Δ* cells, they functionally complemented the respiratory deficiency of the double mutant strain at the same level as the *ctr4^+^* and *ctr5^+^* wild-type, untagged alleles (Fig. 2 and data not shown). This important difference between the *S. pombe* Ctr4–Ctr5 copper transport system and *S. cerevisiae* Ctr1 supports the existence of distinct mechanisms for transporter regulation between different family members.

At the present state of knowledge, the molecular process by which the Ctr4–Ctr5 copper transport complex is stimulated to undergo internalization and recycling in response to changes in copper levels is unknown. We suggest that there are specific signals in either Ctr4 or Ctr5, and additional cellular factors, that are required for both the internalization of the Ctr4–Ctr5 complex and its return to the plasma membrane. It has been shown that the copper-regulated subcellular localization and transport function of the human ATP7A/ATP7B copper ATPases and hCTR1 are mechanistically coupled [1]. Consequently, it is tempting to speculate that the Met-rich domains of Ctr4 and the Ctr4 residue Met-122, which have been shown to play important roles in copper assimilation [22], may also participate in the copper-stimulated trafficking response of the heteromeric Ctr4–Ctr5 complex. Similarly, the Met-rich domains of Ctr5 and the Ctr5 Cys-X-Met-X-Met sequence may also be required for the post-translational regulation of the Ctr4–Ctr5 complex [22]. The fact that the sequence identity between Ctr4 and Ctr5 is relatively low may signify that there are distinct mechanistic contributions from both Ctr4 and Ctr5 in the copper-regulated trafficking of the heteromeric protein complex. For example, either Ctr4 or Ctr5 may participate in molecular interactions with other partners that play a critical role in both signaling and either endocytic or exocytic movement in response to changes in copper levels. Future studies will be needed to establish their respective mechanistic contributions.

## Materials and Methods

### Strains and media

The *S. pombe* strains used in this study were the wild-type FY435 (*h+ his7-366 leu1-32 ura4-Δ18 ade6-M210*) and the *ctr4Δ ctr5Δ* double mutant disruption strain (isogenic to FY435 plus *ctr4Δ::ura4^+^ ctr5Δ::KAN^r^*) [22]. Under non-selective conditions, *S. pombe* cells were grown in yeast extract plus supplements (YES) containing 225 mg/l of adenine, histidine, leucine, uracil and lysine. The fermentable carbon source YES medium contains 3% glucose, whereas the non-fermentable carbon source YES-glycerol/ethanol medium was prepared by replacing the glucose in YES with a combination of 3% glycerol (v/v) and 2% ethanol. When plasmid maintenance was necessary, the cells were grown in Edinburgh minimal medium (EMM) lacking the specific amino acids required for plasmid selection [39]. Unsupplemented EMM contained 160 nM copper, unless otherwise stated. To test the ability of transformed cells to grow on either a fermentable or a non-fermentable carbon source, the indicated cells were grown to mid-logarithmic phase and then spotted onto agar media containing either glucose, or glycerol/ethanol with 0, 5, 10, 20 or 25 µM of the copper chelator bathocuproine disulfonic acid (BCS). Spotted cells were grown either for 5 days on media containing glucose, or for 9 days on media containing glycerol/ethanol either with or without BCS.

### *Ctr4* plasmids

To generate the pBP*ctr4^+^-VC* plasmid, a BamHI-SacI polymerase chain reaction (PCR)-amplified DNA segment containing the nonfluorescent C-terminal (VC) coding fragment of Venus was isolated from the pFA6a-VC155-KANMX6 plasmid [25] and then inserted into the BamHI and SacI sites of the plasmid pBP*ctr4^+^* [22]. A similar strategy was used to create the plasmid pBP*ctr4^+^-VN*, except that a BamHI-SacI PCR-amplified DNA segment containing the nonfluorescent N-terminal (VN) coding fragment of Venus was isolated from the pFA6a-VN173-KANMX6 plasmid [25]. The pBP*ctr4^+^-VN* plasmid was digested with ApaI and SacI to isolate the *ctr4^+^-VN* fusion coding sequence, which was subsequently cloned into the same restriction sites in plasmid pJK148 [28]. The resulting plasmid was named pJK*ctr4^+^-VN*. The plasmid pBP*ctr4^+^-Flag_2_* was constructed by isolating the SacI-SalI fragment from pSP*ctr4^+^-Flag_2_* [21]. This DNA restriction fragment contained both the *ctr4^+^-Flag_2_* fusion gene and its promoter region up to 737 nucleotides upstream of the initiator codon of the *ctr4^+^* gene.

### *Ctr5* plasmids

To create a plasmid that had the *ctr5^+^* gene coupled with twelve copies of the *Myc* epitope, the DNA fragment containing the *ctr5^+^* gene was isolated from the pSK*ctr5^+^* plasmid [22] using PstI and XmaI (isoschizomer of SmaI), and was then inserted into the corresponding sites of p*ctr4^+^-X-Myc_12_* [40]. By substituting the *ctr5^+^* promoter in place of the *ctr4^+^* promoter, the plasmid had the *ctr5^+^* promoter driving the expression of the *ctr5^+^-Myc_12_* fusion gene. Once created, the *ctr5^+^-Myc_12_* fusion gene and its regulatory region were isolated using PstI and XbaI, and were then inserted into the corresponding sites of pJK148. The resulting plasmid was designated pJK*ctr5^+^-Myc_12_*. Subsequently, using Asp718 and NotI (enzymes with restriction sites that are located on either side of the insert), the *ctr5^+^-Myc_12_* fusion gene was cloned into the Asp718/NotI-cut pEA500 to generate pEA*ctr5^+^-Myc_12_*. Next, using Asp718 and SacI, the *ctr5^+^-Myc_12_* fusion gene was inserted into an Asp718/SacI-cut pSP1 to create pSP1*ctr5^+^-Myc_12_*. To generate the pSP*ctr5^+^-VN* plasmid, an XmaI-SacI PCR-amplified DNA segment containing the VN coding fragment was isolated from the plasmid pFA6a-VN173-KANMX6 [25] and then substituted for the XmaI-SacI restriction fragment present in the plasmid pSP1*ctr5^+^-Myc_12_*, thereby replacing the Myc epitope with the VN fragment. To create a plasmid possessing the *ctr5^+^* gene in-frame with the VC coding fragment of Venus, the *ctr5^+^* gene and its promoter region was isolated from pSP1*ctr5^+^-VN* by Asp718 and XmaI digestions. The purified Asp718-XmaI DNA fragment was then cloned into the corresponding sites of pEA500 [41], generating pEA*ctr5^+^*. The coding sequence of VC was PCR amplified from pFA6a-VC155-KANMX6 [25] and then inserted into the XmaI and BamHI restriction sites of pEA*ctr5^+^*, creating pEA*ctr5^+^-VC*.

### Microscopic analysis of BiFC signals

*ctr4Δ ctr5Δ* double mutant cells co-expressing BiFC fusion proteins were grown in EMM medium containing the appropriate amino acids (225 mg/liter) for the BiFC system selection. The cells were grown to an *A_600_* of ∼0.5. These mid-logarithmic cells were then incubated in the presence of CuSO_4_ (1, 25 or 100 µM), FeCl_3_ (100 µM), or BCS (100 µM). After a 3 h treatment, direct fluorescence images for BiFC signal were taken using a fluorescein isothiocyanate filter set (excitation band pass filter, 465–495 nm; beam splitter, 505 nm; emission band pass filter, 515–555 nm). Microscopy was performed with a Nikon Eclipse E800 epifluorescent microscope (Nikon, Melville, NY) equipped with a Hamamatsu ORCA-ER cooled CCD camera (Hamamatsu, Bridgewater, NJ). The cell fields shown in this study are representative of at least five independent experiments.

### Inhibition of protein synthesis

To terminate cytoplasmic translation, mid-logarithmic cells (*A_600_* of ∼0.5) were treated with cycloheximide (100 µg/ml) for 30 min prior to addition of either copper or BCS. Because the growth rate of cycloheximide-treated cells was inhibited, as compared with control cells, it was considered that *de novo* protein synthesis had been blocked by cycloheximide in an effective manner.
